# Genome Sequence of *Dengue virus 3* from the *Pythium insidiosum* Transcriptomes

**DOI:** 10.3389/fmicb.2016.00926

**Published:** 2016-06-15

**Authors:** Yeonhwa Jo, Hoseong Choi, Won K. Cho

**Affiliations:** ^1^Department of Agricultural Biotechnology, College of Agriculture and Life Sciences, Seoul National UniversitySeoul, Republic of Korea; ^2^The Taejin Genome InstituteHoengseong, Republic of Korea

**Keywords:** genome, *Dengue virus 3*, *Pythium insidiosum*, *de novo* assembly, single nucleotide polymorphism

## Background

*Pythium* species are a kind of the fungus-like oomycetes presenting in soil and aquatic environments (Latijnhouwers et al., 2003). They are regarded as serious plant pathogens, resulting in economic losses of many crops. Of known *Pythium* species, *P. insidiosum* is the only oomycete that can infect both humans and animals (Gaastra et al., 2010). *P. insidiosum* causes a life-threatening infectious diseases known as pythiosis (Mendoza et al., 1993; Gaastra et al., 2010). So far, infection of *P. insidiosum* has been reported in horses, dogs, and humans in tropical and subtropical regions. *P. insidiosum* inhabits swampy areas and produces motile zoospores colonizing surfaces of water plants (Mendoza et al., 1993; Gaastra et al., 2010). Contact of zoospores with tissues of human and animal causes serious infection (Mendoza et al., 1993).

Dengue virus (DENV) causes severe visceral and central nervous system disease in humans (Martina et al., 2009). DENV belongs to the genus *Flavivirus* and consists of single-stranded positive-sense RNA (Chambers et al., 1990; Bai et al., 2013). Infection of DENV is mediated by two major mosquito vectors: *Aedes albopictus* and *Aedes aegypti* (Martina et al., 2009). So far, four different serotypes of DENV have been identified in tropical and subtropical regions (Bai et al., 2013). The genome of DENV is composed of a single-stranded positive-sense RNA about 10,700 nucleotides (nt) in length. The DENV genome consists of a single open reading frame (ORF) encoding three structural (C, prM, and E) and seven nonstructural (NS1, NS2A, NS2B, NS3, NS4A, NS4B, and NS5) proteins (Chambers et al., 1990).

Next generation sequencing (NGS) facilitates the identification of known viruses in various organisms (Radford et al., 2012). NGS can be applied not only in detection or diagnosis of viruses but also in metagenome-based approaches to detect unexpected disease-associated viruses and novel viruses (Adams et al., 2009; Radford et al., 2012). In addition, complete or draft viral genomes can be assembled from NGS data (Jo et al., 2015).

In this study, we identified DENV3 from *P. insidiosum* transcriptome data and assembled a nearly complete genome of DENV3. In addition, we examined single nucleotide variations (SNVs) in the *P. insidiosum* transcriptome, demonstrating quasispecies of DENV3.

## Methods

### Oomycete material, growth condition, and library preparation for 454 sequencing

The *P. insidiosum* transcriptome data was obtained from the previous study (Krajaejun et al., 2014). In brief, *P. insidiosum* strain Pi-S was isolated from a Thai patient showing vascular pythiosis. The obtained *P. insidiosum* was cultured in Sabouraud dextrose broth and incubated at 28°C and 37°C for 1 week. Mycelia from each condition was harvested and washed with sterile water. Again, the mycelia samples were transferred to 2 ml of microcentrifuge tube and incubated at 28°C and 37°C, respectively, for 24 h. Harvested mycelia was immediately frozen in liquid nitrogen for further study. Poly-A tailed mRNAs were isolated from total RNAs and used for cDNA library preparation. Sequencing was performed using the Genome Sequencer (GS) FLX Titanium platform (Roche Applied Sciences, Penzberg, Germany).

### *De novo* transcriptome assembly

We downloaded two raw data with following accession numbers, DRR004443 and DRR004444, from DDBJ (DNA Data Bank of Japan) (https://trace.ddbj.nig.ac.jp/index_e.html). We used a workstation (two six-core CPUs and 256-GB RAM) operated using the Ubuntu 12.04.5 LTS operation system for all bioinformatics analyses in this study. Each individual FASTQ file for each library was first subjected to *de novo* transcriptome assembly using the Trinity program (version 2.0.2, released 22nd January 2015) with default parameters (Grabherr et al., 2011). We obtained two independent transcriptomes from the two samples (Table 1). For *de novo* genome assembly of DENV3, we combined two raw data sets and performed transcriptome assembly again using Trinity, Velvet programs (Zerbino and Birney, 2008), and iAssembler program (Zheng et al., 2011). After that, contigs associated with DENV3 were selected by blast search against known DENV3 reference sequences. Genome of DENV3 was manually assembled.

**Table 1:** **Summary of ***de novo*** transcriptome assembly and the number of identified DENV3 associated reads and contigs**.

**Index**	**28°C**	**37°C**
SRA accession No.	DRR004443	DRR004444
Total genes	14,327	17,761
Total transcripts	14537	18049
Percent GC	59.23	60.29
Contig N50	794	827
Median contig length	697	726
Average contig	751.7	797.18
Total assembled bases	10927464	14388240
Total No. of reads	177947	204241
No. of DENV3 reads	638	229
No. of DENV3 contigs	116	20

### Identification of viruses in the *P. insidiosum* transcriptomes

To identify viruses in the *P. insidiosum* transcriptomes, two different approaches were used. For the first, *de novo* assembled contigs were blasted against complete viral reference sequences (http://www.ncbi.nlm.nih.gov/genome/viruses/). For the BLAST search, the MEGABLAST algorithm with a cut-off *e*-value of 1e-5 was applied. MEGABLAST is much faster than other sequence similarity programs and provides very reliable sequence similarities for the identification of viruses. For the second approach, all the FASTQ files were converted into FASTA files using the FASTX-Toolkit (http://hannonlab.cshl.edu/fastx_toolkit/) and FASTA files from each library were directly blasted against complete viral reference sequences.

### Examination of single nucleotide variations

To identify SNVs, all the raw data (FASTQ files) were aligned on the obtained DENV3 genome sequence using the Burrows–Wheeler Aligner (BWA) with default parameters (Li and Durbin, 2009). The obtained SAM files were converted to BAM files for sorting using SAMtools (Li et al., 2009). The BAM files were sorted for SNV calling using SAMtools. Afterward, mpileup was conducted to generate the VCF format. The SNVs were then called using BCFtools implemented in SAMtools, and finally, SNVs were filtered using BCFtools. For the visualization of mapped reads on the genome, aligned SAM files were imported to the Tablet program (Milne et al., 2010).

## Results

### Identification of DENV3 in the *P. insidiosum* transcriptomes

**Figure 1:**
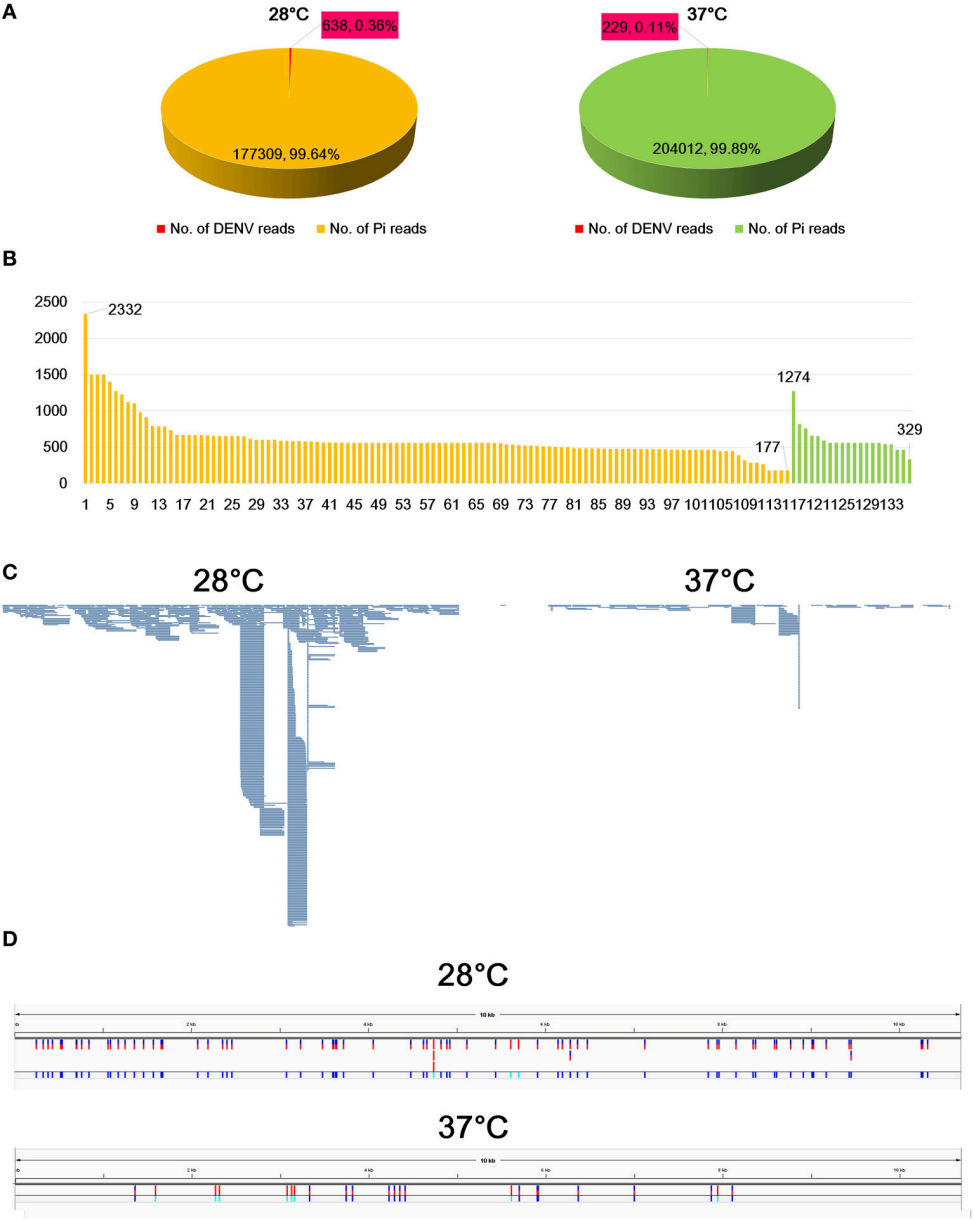
**Identification of sequences associated with DENV3 from ***P. insidiosum*** transcriptome data. (A)** The number of reads associated with DENV3 in samples grown at 28°C and 37°C indicated by red colored box. **(B)** The length distribution of identified contigs associated with DENV3. Orange and green colored bars indicate samples grown at 28°C and 37°C, respectively. The number indicates the length of contigs (bp) **(C)** Mapped DENV3 associated with reads on the assembled DENV3 genome, which was visualized by the Tablet program. **(D)** The locations of identified SNVs along the DENV3 genome in two samples grown at 28°C and 37°C, respectively.

In a search of viruses infecting fungi in the publicly available transcriptome data, we found DENV3-associated sequences from *P. insidiosum* transcriptomes. The transcriptomes were composed of two different libraries, which were generated from two different growth conditions, 28°C and 37°C. BLAST search revealed that 0.36% (638 reads) and 0.11% (229 reads) at 28°C and 37°C, respectively, were sequences associated with DENV3 (Figure 1A). The portion of DENV3 associated reads at 28°C was more than three times that at 37°C. This result indicates that replication of DENV3 was reduced two-fold when the temperature was increased.

### *De novo* viral genome assembly for DENV3

Many sequenced reads were associated DENV3. Therefore, we *de novo* assembled transcriptome of *P. insidiosum* to identify contigs associated with the DENV3 genome. BLAST search identified 116 (28°C) and 20 (37°C) contigs associated with DENV3 (Figure 1B; Supplementary Table S1). The longest contig was 2332 bp, while the shortest contig was 177 bp. We assembled the genome of DENV3. The genome of DENV3, referred to as isolate Pythium, was 10,671 nucleotides (nt) in length (accession number: KT424097). By BLASTN search against the NCBI nucleotide database, the DENV3 isolate Pythium was found to be highly matched to the known DENV3 isolate HN/2013/20 (KJ622192.1) with 98% sequence identity. The DENV3 isolate HN/2013/20 was isolated from Henan, China, in 2013, indicating the possible origin of DENV3 isolate Pythium.

We mapped raw data on the genome of the obtained DENV3 isolate Pythium. As shown in Figure 1C, the number of reads mapped on the genome was much higher in the transcriptome from 28°C as compared to that of 37°C (Figure 1C).

### Analysis of single nucleotide variations

We examined SNVs for DENV3 in two different transcriptomes (Supplementary Table S2). From the transcriptome at 28°C, we identified 73 SNVs including 11 InDels (Insertion and Deletions), while 23 SNVs harboring nine InDels were detected from the transcriptome at 37°C (Figure 1D). This data strongly supports the quasispecies nature of DENV3 within the *P. insidiosum* host (Kurosu, 2011).

## Discussion

The host ranges of viruses were numerous, from single cellular organisms to multicellular organisms. Previously, several viruses have been identified in many oomycetes, including *Phytophthora* and *Pythium* species (Gillings et al., 1993; Cai and Hillman, 2013). In addition, double-stranded RNA virus has been identified in the human pathogenic fungus *Blastomyces dermatitidis* (Kohno et al., 1994). A recent study using electron microscopy demonstrated that several human pathogenic fungi were infected by virus-like particles (Sharma et al., 2011). However, to our knowledge, no study has clearly identified a virus in the human pathogenic fungus so far.

To clearly demonstrate the presence of DENV3 in *P. insidiosum* transcriptomes, we assembled the genome of DENV3 isolate *Pythium* and examined its SNVs using *Pythium* transcriptome data. However, we are not sure whether the *P. insidiosum* was really infected by DENV3. It seems that the contamination rate of DENV3 in *P. insidiosum* culture should be very high. For example, a patient was infected with dengue virus and had viremia. Total RNAs from the viremia were transferred into *P. insidiosum* culture medium and slowly degraded during culture. Libraries were prepared from the culture medium containing dengue virus and sequenced by a next-generation sequencer. Moreover, the low amount of DENV3 at 37°C as compared to that at 28°C indicates the instability of the DENV3 RNA genome at higher temperatures. If DENV3 can replicate in *P. insidiosum*, we can get a high level of DENV3 RNA genome at 37°C, because flavivirus polymerase usually works better at higher temperatures (Simon et al., 2015).

Many previous studies have also reported microbial contamination in clinical samples by NGS. For example, substantial bacterial contamination has routinely been identified in RNA-Seq data (Strong et al., 2014) as well as DNA data (Laurence et al., 2014). Due to the high level of sensitivity of the NGS technique, NGS is currently used to discover new viruses associated with diseases in clinical virology (Datta et al., 2015; Perlejewski et al., 2015). Taken together, this study provides the complete genome of DENV3 from *P. insidiosum* transcriptomes that might have been contaminated during sample preparation.

## Data access

The two raw data in this study can be available from DDBJ (DNA Data Bank of Japan) (https://trace.ddbj.nig.ac.jp/index_e.html) with following accession numbers, DRR004443 and DRR004444. Genome sequence of DENV3 isolate Pythium was deposited in GenBank with accession number KT424097.

## Author contributions

WC designed the research; YJ, HC, and WC performed the research; YJ, HC, and WC analyzed the data; and YJ, HC, and WC wrote the paper.

### Conflict of interest statement

The authors declare that the research was conducted in the absence of any commercial or financial relationships that could be construed as a potential conflict of interest.

## Supplementary material

Supplementary Table S1 **Detailed information of megablast results to identify contigs associated with DENV3**.

Supplementary Table S2 **Information of identified single nucleotide variations for DENV3 in two samples grown at 28°C and 37°C, respectively**.
